# Cell Fate and Differentiation of Bone Marrow Mesenchymal Stem Cells

**DOI:** 10.1155/2016/3753581

**Published:** 2016-05-19

**Authors:** Shoichiro Kokabu, Jonathan W. Lowery, Eijiro Jimi

**Affiliations:** ^1^Division of Molecular Signaling and Biochemistry, Department of Health Improvement, Kyushu Dental University, 2-6-1 Manazuru, Kokurakita-ku, Kitakyushu, Fukuoka 803-8580, Japan; ^2^Department of Oral and Maxillofacial Surgery, Faculty of Medicine, Saitama Medical University, 38 Morohongo, Moroyama-machi, Iruma-gun, Saitama 350-0495, Japan; ^3^Division of Biomedical Science, College of Osteopathic Medicine, Marian University, 3200 Cold Spring Road, Indianapolis, IN 46222, USA

## Abstract

Osteoblasts and bone marrow adipocytes originate from bone marrow mesenchymal stem cells (BMMSCs) and there appears to be a reciprocal relationship between adipogenesis and osteoblastogenesis. Alterations in the balance between adipogenesis and osteoblastogenesis in BMMSCs wherein adipogenesis is increased relative to osteoblastogenesis are associated with decreased bone quality and quantity. Several proteins have been reported to regulate this reciprocal relationship but the exact nature of the signals regulating the balance between osteoblast and adipocyte formation within the bone marrow space remains to be determined. In this review, we focus on the role of Transducin-Like Enhancer of Split 3 (TLE3), which was recently reported to regulate the balance between osteoblast and adipocyte formation from BMMSCs. We also discuss evidence implicating canonical Wnt signalling, which plays important roles in both adipogenesis and osteoblastogenesis, in regulating TLE3 expression. Currently, there is demand for new effective therapies that target the stimulation of osteoblast differentiation to enhance bone formation. We speculate that reducing TLE3 expression or activity in BMMSCs could be a useful approach towards increasing osteoblast numbers and reducing adipogenesis in the bone marrow environment.

## 1. Introduction

**Figure 1:**
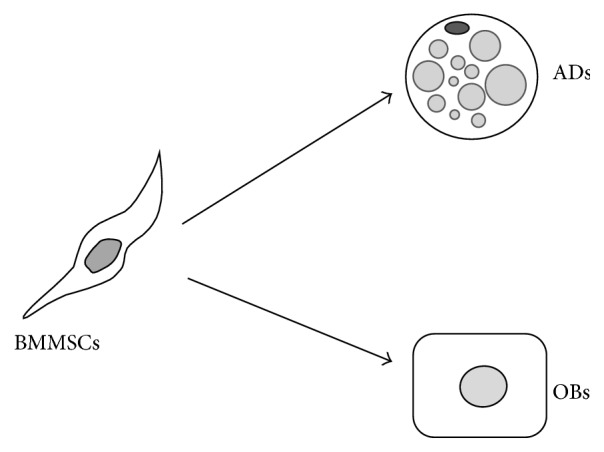
Bone marrow mesenchymal stem cells differentiate into both adipocytes and osteoblasts. Osteoblast and marrow adipocytes are derived from common progenitors, the bone marrow mesenchymal stem cells. BMMSCs: bone marrow mesenchymal stem cells; OBs: osteoblasts; ADs: adipocytes.

In 2010, more than 10 million Americans over the age of 50 had osteoporosis with another 43 million Americans at risk for the disease [1]. It is estimated that greater than 1.5 million fragility fractures occur each year, with an annual health care cost of at least 14 billion US dollars [2]. By 2025, the health care expenditures for osteoporotic fractures will approach 25.3 billion US dollars [3]. Bone is constantly remodeled through the processes of bone formation by osteoblasts and bone resorption by osteoclasts. Osteoclasts are derived from hematopoietic stem cell precursors of the monocyte/macrophage lineage located in the blood and bone marrow [4]; conversely, osteoblast-lineage cells (osteoblasts and osteocytes) originate from bone marrow mesenchymal stem cells (BMMSCs) [5]. BMMSCs are a multipotent cell type that can give rise not only to osteoblast-lineage cells but also to a range of other cell types, including adipocytes [6] (Figure 1). In some pathological conditions, including senile osteoporosis, the balance between adipocyte and osteoblast differentiation is disrupted in this cell population such that adipocyte differentiation is increased relative to osteoblast differentiation and this is associated with reduced bone mass, increased bone fragility, and increased susceptibility to fracture [7]. Therefore, understanding the molecular mechanism(s) responsible for controlling the balance between osteoblastogenesis and adipogenesis in the adult bone environment is of great significance.

In this review, we will summarize the processes of osteoblast and adipocyte differentiation from BMMSCs, focusing on the role of Transducin-Like Enhancer of Split 3 (TLE3), which was recently reported to regulate osteoblastogenesis and adipogenesis. We also discuss the prospect of bone regenerative therapy by using stem cells.

## 2. Relationship between Adipogenesis and Osteoblastogenesis

Adipogenesis is driven by a complex and well-orchestrated signalling cascade composed of several key transcription factors, most notably proliferator-activated receptor- (PPAR-) *γ* and several members of the CCAAT/enhancer-binding family of proteins (C/EBPs) [8]. PPAR-*γ* is commonly referred to as the master regulator of adipogenesis because no factor has yet been identified that can induce normal adipogenesis in its absence [9].

BMP-SMAD signalling plays an important role in osteoblastogenesis by inducing expression of several critical transcription factors such as RUNX2, Osterix, DLX2, and DLX5 [10–12]. RUNX2 is essential for the commitment of mesenchymal stem cells to the osteoblast lineage and homozygous deletion of* Runx2* in mice results in a complete lack of osteoblasts [13, 14]. It appears that adequate RUNX2 is also dosage-dependent since haploinsufficiency of* Runx2* in mice or* RUNX2* in humans causes hypoplastic clavicles and delayed closure of the fontanelles, defects that are characteristic of cleidocranial dysplasia in humans [15, 16]. RUNX2 controls osteoblast-related genes such as* Osterix*,* collagen I*, and* osteocalcin* [17] and autoregulates the* Runx2* gene itself [18].

Several proteins have been reported to regulate both adipogenesis and osteoblastogenesis (Table 1) and, in general, adipogenesis is reciprocally related to osteoblastogenesis in BMMSCs. However, the exact nature of the signals regulating the balance between osteoblast and adipocyte formation within the bone marrow space remains to be determined. In the sections below, we seek to bring attention to TLE3, which is a relatively understudied regulator of osteoblastogenesis and adipogenesis that is a member of the Groucho/TLE family of transcription factors [19].

## 3. Groucho/TLE Family Member

Groucho (Gro)/Transducin-Like Enhancer of Split (TLE) family members are transcriptional cofactors in metazoans that play critical roles during development and cell fate determination, including differentiation into fat and bone cells. The names “Gro” and “TLE” are used interchangeably in the literature and in sequence databases [20] and the* Drosophila* genome encodes a single Gro while the mouse and human genomes encode four members of each family [21].

Groucho/TLE proteins consist of a five-domain structure [22]: a highly conserved Q domain, which is a glutamine-rich region predicted to form two coiled-coil motifs that facilitates oligomerization of Gro/TLE molecules* in vitro* [23–25]; a glycine/proline rich (GP) domain, which is essential for interaction of Groucho/TLE proteins with histone deacetylases (HDACs) [23, 24, 26, 27]; a CcN domain, which contains a nuclear localization sequence and putative cdc2 and casein kinase II (protein kinase CK2) phosphorylation sites; a serine/proline rich (SP) domain, which is a region rich in serine/proline residues [22, 28–30]; and a highly conserved WD40 domain, which contains multiple tryptophan and aspartic acid tandem repeats, has been shown by X-ray crystallography to form a *β*-propeller, and binds many kinds of transcriptional factors [20, 31].

Groucho/TLE proteins do not bind DNA directly but are instead recruited by other transcription factors and are largely considered transcriptional corepressors since they often reduce the activity of a target transcriptional factor. However, the Groucho/TLE family member TLE3 was recently reported to induce the transcriptional activity of PPAR-*γ*, which is a master transcriptional regulator of adipogenesis [32], suggesting that the Groucho/TLE family may act as corepressors or coactivators in a context-dependent manner.

## 4. Distribution of TLE3 during Development

During development, TLE3 is expressed in the placenta [33] and homozygous null* Tle3* mutant mice are smaller than their heterozygous and wild type littermates. Most homozygous null* Tle3* mutant embryos demonstrate severe placental defects and die* in utero* [34]. TLE3 is also expressed in the developing nervous system where as the neural tube closes, its distribution shifts from the entire width of the neural plate to the dorsal region and ventricular zone; expression in the roof of the mesencephalon and metencephalon remains most pronounced at this stage. TLE3 is also expressed in the dorsal root ganglia and its expression in the newly formed somites becomes restricted to a dorsal, bracket-shaped group of cells corresponding to the dermamyotome [35].

In older mouse embryos expression of TLE3 in the central nervous system (CNS) is observed along the entire length of the brain and spinal cord in the ventricular zone, with the strongest expression in the layer of cells immediately lining the lumen. In the developing eye, TLE3 is located in the lens and the neural layer of the retina. Somatic expression of TLE3 continues in the dermamyotome and in the condensing sclerotome, forming the vertebrae and bones. Faint staining for TLE3 is also observed in the metanephros (embryonic kidney); tissues derived from the pharynx, including Rathke's pouch and the thymic primordial; the lining of the gut and tissues derived from the gut endoderm such as the epithelial walls of the bronchi of the lungs and the liver; and derivatives of the branchial arches such as the dorsum and intrinsic muscles of the tongue and the dental laminae of the tooth primordial [35].

In later stages of mouse development (16.5 days after conception), TLE3 expression is more restricted than at midgestation. For instance,* Tle3* mRNA is detected in the ventricular zone and the cortical plate of the cerebral cortex; the colliculus; the cerebellum; the olfactory lobe; nasal epithelia; whisker follicles primordia; epithelial cells of the salivary glands; basal layer of skin and hair follicles; and derivatives of the pharyngeal pouches including the lining of the cochlea, eustachian tube, esophagus, larynx, epiglottis, and the thymus [35]. TLE3 is also expressed by cells of the bone marrow [19] and brown and white adipose tissue [32], with the expression level of TLE3 increasing with adipocyte differentiation [19, 32].

## 5. TLE3 Enhances Adipocyte Differentiation and Suppresses Osteoblastogenesis

Adipocytes are classically classified into two kinds: white adipocytes and brown adipocytes. White adipocytes are optimized to store energy as triglycerides in large, unilocular lipid droplets. When metabolic needs arise, white adipocytes mobilize energy through hydrolysis of triglycerides and release of free fatty acids into the circulation [36]. White adipocytes express a battery of genes involved in lipid handing, triglyceride biosynthesis, triglyceride mobilization, and endocrine signalling [37–39].

Brown adipocytes derive their color from their high mitochondrial content. Unlike white adipocytes, brown adipocytes store energy primarily to provide an intracellular fuel source for thermogenesis [40]. During cold exposure, brown adipose tissue (BAT) executes a transcriptional program that promotes energy expenditure and thermogenesis. Induction of the gene encoding Mitochondrial Uncoupling Protein-1 (UCP1) is critical for brown fat thermogenesis [41, 42]. It has been thought that Ucp1 expression is restricted to BAT; however, recent studies have demonstrated that Ucp-1-positive cells can be detected even in white adipose tissue under certain circumstances. These cells are called “beige adipocytes” [43] and have characteristics of both white and brown adipose cells: during basal state, beige adipocytes display unilocular morphology similar to white adipocytes, but upon cold stimulation, these cells acquire features of intermediate morphology ultimately resulting in expression of proteins typical for BAT and transformation of stored fat into the small lipid droplets typical for brown adipocytes [44–46].

While the transcriptional determinants of the white and brown adipocyte gene programs are incompletely understood, it is known that PPAR*γ* is the master transcriptional regulator of both white and brown fat differentiation. In support of this, mice deficient in PPAR*γ* lack both types of adipose tissue [9, 47–49]. Villanueva et al. [32] identified TLE3 as a cofactor for PPAR*γ* and it was later confirmed that TLE3 enhances transcriptional activity of PPAR*γ*, thereby inducing adipocyte differentiation of BMMSCs [19, 21]. Additionally, TLE3 disrupts the physical interaction between transcriptional cofactor PRDM16, which was identified as a key factor driving brown adipocyte linage development [43, 50], and PPAR*γ*, thereby suppressing brown-fat-specific genes and inducing white-fat-specific genes; the net result of these effects is impaired fatty acid oxidation and thermogenesis [51]. We predict that TLE3 has some influence on beige adipocyte formation, but further studies are needed to examine this possibility.

Described above, osteoblast-lineage cells and marrow adipocytes are derived from a common progenitor, the BMMSCs. RUNX2 controls osteoblast-related genes and is essential for commitment to the osteoblast lineage [13, 14, 52]. RUNX2 interacts with Groucho/TLE family members, which act as corepressors of RUNX2 activity [53, 54]. For instance, TLE1 and TLE2 repress RUNX2-dependent activation of* osteocalcin* gene transcription [55]. And TLE3 suppresses BMP2-induced osteoblast differentiation of BMMSCs via recruiting HDAC and repressing RUNX2 transcriptional activity [19].

## 6. Expression of TLE3 Is Regulated by Canonical Wnt Signalling

The Wnt family of nineteen secreted glycoproteins has a critical role in regulating embryonic development, cell differentiation, and cell fate determination [56]. Wnts transduce two types of intracellular signalling referred to as canonical and noncanonical pathways. Canonical Wnt signalling, that is, signalling mediated by the effector *β*-catenin, has a key role in adult skeletal homeostasis and bone remodeling [57] by promoting differentiation and maturation of osteoblasts and, thereby, increasing bone formation [58]. In contrast, canonical Wnt signalling suppresses adipocyte differentiation [59].

Groucho/TLE family members, including TLE3, act as transcriptional corepressors of canonical Wnt signalling via binding to the downstream effectors TCF/LEF and inhibiting Wnt target gene transcription [20, 60–62]. According to Daniels and Weis [62], *β*-catenin that enters the nucleus upon activation of the Wnt pathway directly competes with Groucho/TLE proteins for TCF/LEF binding to accomplish gene regulation.

**Figure 2:**
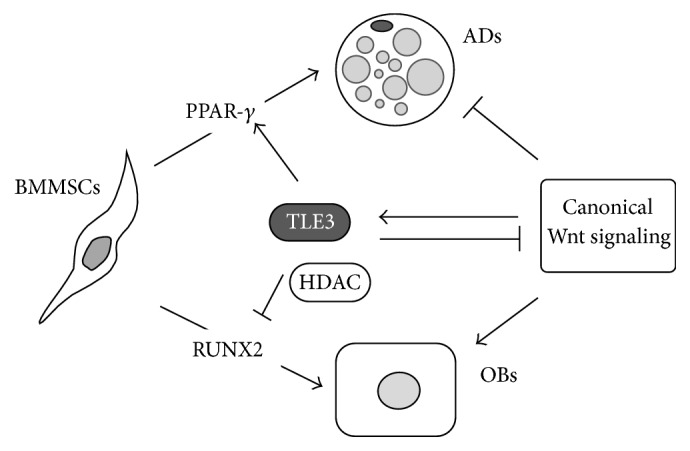
Model for the role of TLE3 in the bone marrow microenvironment. TLE3 directly induces adipogenesis and suppresses osteoblastogenesis of BMMSCs by acting on PPAR-*γ* and RUNX2, respectively. TLE3 also indirectly induces adipogenesis and suppresses osteoblastogenesis by repressing canonical Wnt signalling, which is capable of inducing osteoblastogenesis and inhibiting adipogenesis. In addition, canonical Wnt signalling induces TLE3 expression, suggesting that the induction of TLE3 by Wnt signalling may be part of a negative feedback loop during osteoblastogenesis and/or a positive feedback loop during adipogenesis in the adult bone marrow microenvironment. BMMSCs: bone marrow mesenchymal stem cells; OBs: osteoblasts; ADs: adipocytes.

Recently, Wnt responsive elements in the TLE3 promoter region were identified through comparative genomic analysis and functional analyses confirmed that expression of TLE3 is increased by Wnt signalling [21]. Given the opposing roles of TLE3 and Wnt signalling in BMMSCs differentiation, this finding suggests that induction of TLE3 by Wnt signalling is part of a negative feedback loop active during osteoblast differentiation and/or a part of a positive feedback loop during adipogenesis, suggesting that TLE3 regulates the cell fate of BMMSCs between osteoblasts and adipocytes (Figure 2).

## 7. Prospects for Therapy

Osteoporosis, which is one of the most abundant bone-related diseases, is characterized by low bone mass and microarchitectural deterioration of bone tissue that results in increased bone fragility and susceptibility to fracture [7]. The most commonly prescribed therapeutics are antiresorptives, such as calcitonin, estrogen, and bisphosphonates, that block osteoclast activity as a means to stabilize bone architecture. While efficacious in halting further bone loss, little or no new bone mass is added to the skeleton while on antiresorptive therapy. Recent data on the importance of continuous bone remodeling suggest that overuse of antiresorptives could lead to BRONJ (bisphosphonate-related osteonecrosis of the jaw) [63] and fracture in some patients [64]. Thus, development of new, effective therapies that target enhancing bone formation by stimulating osteoblast differentiation is required.

## 8. Conclusion

In this review we summarized the cell fate determination and the differentiation of BMMSCs and especially focus on the role of TLE3, which represses osteoblast differentiation and enhances adipocyte formation from BMMSCs. Therefore, we speculate that reducing TLE3 expression or activity in BMMSCs could be a useful approach towards increasing osteoblast numbers and reducing adipogenesis in the bone marrow environment. Recently, a delivery system involving dioleoyl trimethylammonium propane- (DOTAP-) based cationic liposomes attached to six repetitive sequences of aspartate, serine, and serine ((AspSerSer)_6_) was utilized to deliver siRNAs specifically to bone formation surfaces [65]. Delivery of siRNAs against* Tle3* with this delivery system might be useful for reducing mRNA levels of TLE3 in bone without affecting other organs and/or tissues. Thus, developing effective methods of reducing TLE3 expression or activity in bone locally may shed light on novel bone formation therapies.

## Figures and Tables

**Table 1:** The proteins regulate adipogenesis and osteoblastogenesis.

Number	Protein(s)	Function	Assay	Reference(s)
1	Msx2	Adipogenesis↓; osteoblastogenesis↑	*In vitro*	[66]
2	Dlk1/Pref-1	Adipogenesis↓; osteoblastogenesis↑	*In vitro*	[67]
3	TAZ	Adipogenesis↓; osteoblastogenesis↑	Zebrafish; *in vitro*	[68]
4	Wnt10b	Adipogenesis↓; osteoblastogenesis↑	Knockout mice; transgenic mice	[69]
5	LIP	Adipogenesis↓; osteoblastogenesis↑	*In vitro*	[70]
6	Dec1	Adipogenesis↓; osteoblastogenesis↑	*In vitro*	[71]
7	Hemooxygenase-1	Adipogenesis↓; osteoblastogenesis↑	*In vitro*	[72]
8	ID4	Adipogenesis↓; osteoblastogenesis↑	Knockout mice	[73]
9	Maf	Adipogenesis↓; osteoblastogenesis↑	Knockout mice	[74]
10	Pkd1	Adipogenesis↓; osteoblastogenesis↑	Knockout mice	[75]
11	sFRP-1	Adipogenesis↑; osteoblastogenesis↓	*In vitro*	[76]
12	ZFP467	Adipogenesis↑; osteoblastogenesis↓	*In vivo injection*	[77]
13	GIT2	Adipogenesis↓; osteoblastogenesis↑	Knockout mice	[78]
14	Wnt6	Adipogenesis↓; osteoblastogenesis↑	*In vitro*	[79]
15	Wnt10a	Adipogenesis↓; osteoblastogenesis↑	*In vitro*	[79]
16	VEGF	Adipogenesis↓; osteoblastogenesis↑	Knockout mice	[80]
17	Semaphorin 3A	Adipogenesis↓; osteoblastogenesis↑	Knockout mice	[81]
18	TLE3	Adipogenesis↑; osteoblastogenesis↓	*In vitro*	[19]
19	S100a16	Adipogenesis↑; osteoblastogenesis↓	*In vitro*	[82]
20	mTORC2	Adipogenesis↓; osteoblastogenesis↑	*In vitro*	[83]
21	Adiponectin	Adipogenesis↓; osteoblastogenesis↑	Knockout mice	[84]
22	Cysteine dioxygenase type 1	Adipogenesis↑; osteoblastogenesis↓	*In vitro*	[85, 86]
23	MYSM1	Adipogenesis↓; osteoblastogenesis↑	Knockout mice	[87]
